# Opium and Stramonium

**Published:** 1868-12-01

**Authors:** James T. Newman

**Affiliations:** Chicago


### OPIUM AND STRAMONIUM.

BY JAMES T. NEWMAN, M.D., CHICAGO.

It has long been a subject of debate in my mind whether or not there existed an antagonism between opium and stramonium; hence, I resolved, on the first opportunity that presented itself, to test them. But, having no chance to experiment, it looked to me as though the problem would never be solved, as far as I was concerned. Yet an unlooked for accident has more than rewarded my patience, and I can say with much pleasure and confidence that one is antagonistic to the other. I feel perfectly satisfied that in opium we have an antidote for belladonna and stramonium. Should cases of poisoning occur from either of those drugs, and the patient seen sufficiently early, there is no need of resorting to them. But, on the other hand, if the poison has been taken up by absorption, we then need an agent to hunt it up and undo what has been done.

To the case in question. Was called on the 1st of October, about half past one o'clock in the morning, to see a mother and her two daughters. They had had paroxysms of ague, and the mother resolved on being her own doctor. She conceived the idea that fennel seed was the medicine she wanted. She sent one of her little girls to a German drug store for the aforesaid fennel seed. Having obtained them, she at once proceeded to make a tea; the strength of it I can not tell. When I was called in, I found the mother and her two children were in a frightful condition. The mother and oldest girl were raving like maniacs, while the youngest girl was rapidly sinking into a state of coma. I soon perceived that they all were laboring under the influence of some powerful narcotic, but what one of them I was unable to determine. I called the attendant, and was informed that she had made a tea of fennel seed, and had drank freely of it herself, and had given the children one or two cups full. I immediately inspected the vessel containing the tea, and to my great horror I found that my patient had been poisoned with stramonium. Two of them were maniacs, and the other almost expiring. What must be done? To think of giving emetics would be out of the question. Hence, I resolved that *Opium* should be their saviour. I sat down and wrote two prescriptions, one for two ounces of *Tincture opii*, and the other for *Morphia sulphatis*, gr. viii., in two ounces *Aqua*. Ordered the mother and girl to have one one teaspoonfull every half hour, and the younger child was treated with the solution mentioned above. The hypodermic syringe was called into use every half hour. I injected beneath the skin two drachms of the liquid. The child recovered rapidly, but the mother and the oldest girl seemed to defy treatment. What must I do? I could not give them up to die! About this time it was daylight. I thought that I would change the *Tincture opii* for *Morphia*. Accordingly I wrote for twelve grains of *Morphia sulphatis*, to be divided into four parts, one every two hours, and at the same time for

℞ *Ammonia, Arom. Spts.*, ℥iij.
*Syr. Zingiberis*, ʒij.
*Misce. fiat mist.*
*Sig.*—Two tablespoonfulls in a wine glass of water every half hour.

The ammonia seemed to give them more strength, but was far from quieting their ravings, and not until the second dose of *Morphia* was given, could I perceive any contraction of the pupils. At twelve o'clock, October 20th, the last dose of *Morphia* was given. Precisely at half past one the young lady fell off into a sleep; the mother soon followed. I ordered every thing to be kept quiet. I called again at five in the evening, but finding they were still asleep; did not disturb them. Called at nine; found them both sitting up in bed, but very weak. The youngest girl was drinking some tea, but not made from the seed of stramonium. The mother and the other girl both refused to eat. Soon went to sleep again; rested quietly all night, and in a few days they were attending to their labors as usual.

In presenting this to your readers, I do not claim any thing new, but there is one thing that I feel assured of: I hav determined the truth of it myself.

